# Identifying a high-risk CKM syndrome phenotype: association of albuminuria with subclinical hemodynamic alterations via portable noninvasive cardiometer

**DOI:** 10.3389/fendo.2026.1966139

**Published:** 2026-09-29

**Authors:** Ting-Chun Chen, Chih-Li Lin, Chien-Yin Kuo, Sing-Hua Tsou, Shun-Wen Hsiao, Chien-Ning Huang

**Affiliations:** 1 Institute of Medicine, Chung Shan Medical University, Taichung, Taiwan; 2 Division of Endocrinology and Metabolism, Cheng Ching Hospital, Taichung, Taiwan; 3 Department of Surgery, Chung Shan Medical University Hospital, Taichung, Taiwan; 4 Department of Medical Research, Chung Shan Medical University Hospital, Taichung, Taiwan; 5 Division of Gastroenterology, Changhua Christian Hospital, Changhua, Taiwan; 6 Department of Post-Baccalaureate Medicine, College of Medicine, National Chung Hsing University, Taichung, Taiwan; 7 Division of Endocrinology and Metabolism, Chung-Shan Medical University Hospital, Taichung, Taiwan

**Keywords:** albuminuria, cardiovascular-kidney-metabolic syndrome, index of contractility, portable noninvasive cardiometry, type 2 diabetes mellitus

## Abstract

**Object:**

Type 2 diabetes mellitus (T2DM) is closely linked to heart failure.

**Methods:**

This prospective study of 274 asymptomatic patients with T2DM without clinical heart failure or coronary artery disease used portable noninvasive cardiometry to assess hemodynamic parameters.

**Results:**

Patients with albuminuria (n=119) showed significantly lower stroke volume and index of contractility (ICON 29.44 ± 9.48 vs 34.78 ± 10.18, p<0.0001), and higher systemic vascular resistance compared with those without albuminuria (n=155). Albuminuria remained independently associated with reduced ICON after multivariable adjustment that included sex, body mass index, and metabolic parameters. Higher BMI was also strongly associated with lower ICON. Patients with albuminuria also had a higher all-cause medical hospitalization rate (39.5% vs 24.5%) during follow-up.

**Discussion:**

These findings suggest that T2DM patients with albuminuria may represent a high-risk CKM phenotype characterized by subclinical hemodynamic alterations, supporting further investigation of noninvasive approaches for early cardiovascular risk assessment.

## Introduction

T2DM is associated with several severe complications. People with diabetes have about twice the risk of HF compared with those without diabetes (1), and incidence is roughly 2–3 times higher in T2DM (2). In a modern Spanish outpatient cohort of T2D, baseline HF prevalence was 39% and 3-year incidence was 7.6% (3.0/100 person-years) (3). These two disorders frequently overlap. Recently, the American Heart Association (AHA) has integrated these interrelated conditions into a single clinical entity known as Cardiovascular-Kidney-Metabolic (CKM) syndrome (4). This framework recognizes the multisystem pathophysiology where metabolic risks, such as T2DM, interact with renal impairment to accelerate cardiovascular dysfunction. Within the CKM spectrum, T2DM and heart failure are deeply patho-physiologically interlinked. Both conditions are characterized by insulin resistance and are accompanied by the activation of neurohormonal systems including norepinephrine, angiotensin II, aldosterone, and neprilysin (5, 6).

The American College of Cardiology (ACC) and American Heart Association (AHA) advocate screening biomarkers, such as N-terminal pro-B-type natriuretic peptide (NT-proBNP), in asymptomatic diabetic patients to detect Stage A (at-risk) or Stage B (preclinical) heart failure, enabling early intervention to prevent progression to Stage C (symptomatic) or Stage D heart failure (Advanced) (5). However, the low specificity of these biomarkers often limits their utility in clinical practice (7). Furthermore, while transthoracic echocardiography is the clinical standard for evaluating cardiac structure and function, it is typically not performed until symptoms manifest (8).

This study included outpatients with T2DM receiving regular clinical care and no clinical evidence of CVD, aiming to explore the early spectrum of Stage A and Stage B heart failure. We utilized a portable noninvasive cardiometer that employs bioimpedance to measure hemodynamic parameters such as stroke volume (SV), cardiac output (CO), and systemic vascular resistance (SVR). This technology has been evaluated against reference methods in several clinical settings, including patients with advanced heart failure and critically ill or surgical populations (9–12). A systematic review and meta-analysis demonstrated acceptable accuracy and precision for cardiac output assessment, supporting its clinical applicability for cardiovascular monitoring (13). By employing this device to gather objective cardiac parameters, we investigated the potential for cardiac impairment in T2DM patients and analyzed the correlation between clinical risk factors and cardiac dysfunction.

## Materials & methods

### Inclusion criteria

We prospectively enrolled patients from November 2018 to November 2019, who were outpatients with T2DM receiving regular clinical care. This study has undergone review and received approval from the Institutional Review Board (IRB) for medical ethics (IRB No: CS18242 and CS16021).

According to American Diabetes Association (ADA), the diagnosis of T2DM was defined as one of the following criteria: HbA1c ≥6.5%(48 mmol/mol) or fasting plasma glucose ≥126 mg/dL or 2 hours plasma glucose ≥200 mg/dL during oral glucose tolerance test or individual with classic symptoms of hyperglycemia or hyperglycemic crisis, with a random plasma glucose ≥200 mg/dL (14). We excluded patients who were diagnosed with heart failure, coronary artery disease or valvular heart disease, pregnancy, dementia, incomplete data or informed consent was not obtained. These patients received standard care for blood sugar control.

### Portable noninvasive cardiometer

We used a portable noninvasive cardiometer (ICON™, Osypka Medical GmbH, Germany) to measure heart rate (HR), CO, [l/min], SV, [ml], index of contractility (ICON), systolic time ratio (STR), stroke volume variation (SVV, [%]), pre-ejection period (PEP, [ms]), left ventricular ejection time (LVET, [ms]), SVR, [Dynes/sec/cm-5], thoracic fluid content(TFC), and corrected flow time(CFT). Each patient was monitored for approximately five minutes, with measurements conducted in a seated position while the patient remained still. A total of six sets of values were measured, and the three consecutive data points with the highest signal quality indicator (SQI) values were selected (SQI represents accuracy, with a maximum value of 100).

### Clinical data extraction

We reviewed the medical records and extracted demographic characteristics and the most recent laboratory results obtained before enrollment, including age, gender, body mass index (BMI), systolic blood pressure (SBP) and laboratory measurements such as glycated hemoglobin (HbA1c), total cholesterol(TC), triglyceride (TG), low density lipoprotein cholesterol (LDL-C), high density lipoprotein cholesterol (HDL-C), urine albumin-creatinine ratio (UACR), creatinine, and estimated glomerular filtration rate(eGFR). Albuminuria was defined as UACR ≥30 mg/g based on a single measurement obtained at enrollment. We traced medical record till November, 2024 and documented patients hospitalized for medical conditions while excluding those admitted for surgical procedures or trauma-related causes.

### Statistical analysis

Demographic and clinical characteristics were presented as frequencies (%), medians with interquartile ranges (IQR, 25th–75th percentile), or means ± standard deviations (SD), as appropriate. The normality of continuous variables was assessed using the one-sample Kolmogorov–Smirnov test. Depending on the data distribution, comparisons of continuous variables were performed using either the Student’s t-test or the Mann–Whitney U test. Categorical variables were analyzed using the chi-square test or Fisher’s exact test, as appropriate. Multiple linear regression was conducted to identify factors independently associated with the index of contractility. A two-sided P-value of <0.05 was considered statistically significant. All statistical analyses were performed using SAS version 9.4 (SAS Institute Inc., Cary, NC, USA).

## Results

### Baseline characteristics

A total of 274 patients with T2DM were included in the final analysis (**Figure 1**). Baseline clinical and laboratory characteristics of the overall cohort and according to albuminuria status are presented in **Table 1**. The mean age was 60.4 ± 9.4 years, and 49.6% were male. Patients with albuminuria (n=119) had significantly higher BMI, SBP, and TG levels, and lower HDL and eGFR compared with those without albuminuria (n=155).

**Figure 1:**
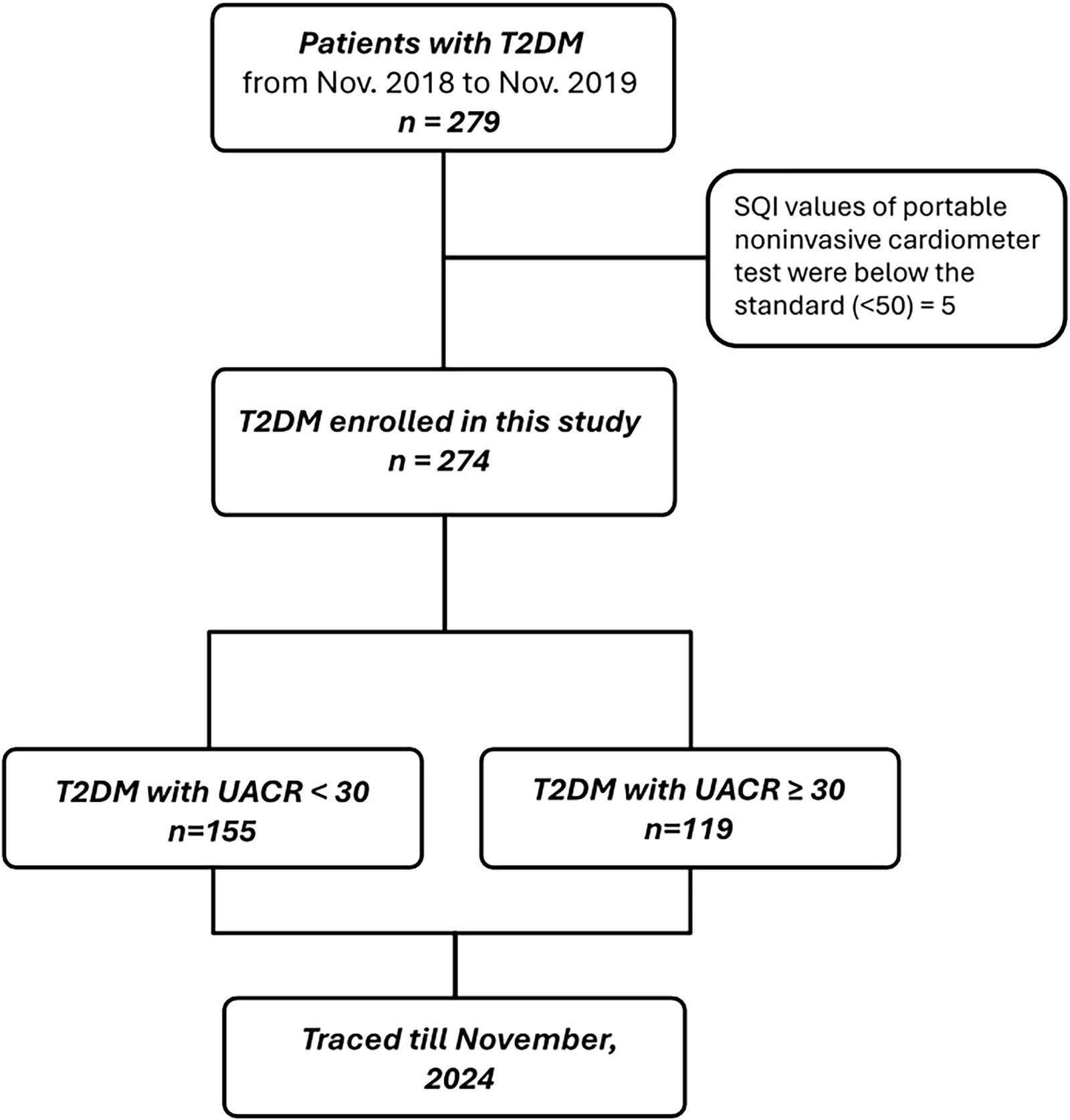
Patient allocation flowchart.

**Table 1:** Baseline characteristics of the study population according to albuminuria status.

Variable	All patients (n=274)	UACR<30 (n=155)	UACR≥30 (n=119)	P value
Age, years, Mean (± SD)	60.43 ± 9.40	59.92 ± 9.26	61.09 ± 9.57	P = 0.3081
Sex (M), n (%)	136 (49.64)	76 (49.03)	60 (50.42)	P = 0.8198
BMI, kg/m2, Mean (± SD)	27.47 ± 4.05	26.76 ± 3.66	28.41 ± 4.36	P = 0.0010
SBP, mmHg, Mean (± SD)	139.99 ± 19.72	137.5 ± 18.09	143.2 ± 21.31	P = 0.0196
HbA1c, %, Median (IQR)	7.3 (6.7-8.0)	7.2(6.7-7.9)	7.4(6.8-8.3)	P = 0.0820
TC, mg/dL, Median (IQR)	171(149.0-195.0)	173.0(148.0-195.0)	170.0(149.0-194.0)	P = 0.9381
LDL-C, mg/dL, Median (IQR)	96(77-115)	96(77.0-116.0)	93(77.0-113.0)	P = 0.4922
TG, mg/dL, Median (IQR)	132 (91-194)	121.0(81.0-170.0)	158.0(96.0-235.0)	P = 0.0002
HDL-C, mg/dL, Median (IQR)	42.7(41.0-44.8)	44.80(37.7-54.0)	39.55(32.6-48.1)	P = 0.0007
Creatinine, mg/dL, Median (IQR)	0.87(0.69-1.01)	0.86(0.68-0.97)	0.89(0.70-1.07)	P = 0.0385
eGFR, mL/min/1.73m^2^, Median (IQR)	89(71-102)	91.0(76.0-104.0)	86.0(62.0-98.0)	P = 0.0191
UACR, mg/g, Median (IQR)	19.35(6.50-65.90)	7.4(5.1-13.1)	79.0(44.7-411.3)	P < 0.0001

SBP, systolic blood pressure, HbA1c, glycated hemoglobin, TC, total cholesterol, LDL-C, low density lipoprotein cholesterol, TG, triglyceride, HDL-C, high density lipoprotein cholesterol, eGFR, estimated Glomerular filtration rate, UACR, urine albumin to creatinine ratio.

### Hemodynamic parameters

Hemodynamic parameters measured by portable noninvasive cardiometry are shown in **Table 2**. The overall cohort exhibited values below typical reference ranges for SV, CO, and ICON, together with elevated SVR.

**Table 2:** Hemodynamic parameters measured by portable noninvasive cardiometry according to albuminuria status.

Variable	All patients (n=274)	UACR<30 (n=155)	UACR≥30 (n=119)	P value	Reference ranges
HR, bpm, Mean (± SD)	85.15 ± 12.36	83.67 ± 12.30	87.08 ± 12.21	P = 0.0231	55-80
CO, l/min, Mean (± SD)	4.36 ± 1.01	4.42 ± 0.97	4.29 ± 1.06	P = 0.2974	4.8-7.2
SV, ml, Mean (± SD)	54.44 ± 10.40	55.84 ± 9.80	52.61 ± 10.91	P = 0.0113	60-130
SVV, %, Mean (± SD)	11.55 ± 4.52	10.65 ± 4.20	12.73 ± 4.66	P = 0.0001	5-10
SVR, Median (IQR)	1811.17(1513.33-2135.33)	1730.33(1493.67-2033.00)	1908.33(1562.00-2237.33)	P = 0.0152	900-1500
LVET, ms, Mean (± SD)	279.84 ± 25.13	281.5 ± 24.42	277.7 ± 25.97	P = 0.2229	280-330
TFC, Mean (± SD)	20.35 ± 5.15	20.36 ± 4.76	20.33 ± 5.63	P = 0.9712	25-35
CFT, Mean (± SD)	322.20 ± 17.73	322.7 ± 15.18	321.5 ± 20.63	P = 0.6059	200-400
ICON, Mean (± SD)	32.45 ± 10.22	34.78 ± 10.18	29.44 ± 9.48	P < 0.0001	40-60
STR, Mean (± SD)	0.42 ± 0.09	0.42 ± 0.09	0.41 ± 0.10	P = 0.6806	0.3-0.5
PEP, ms, Mean (± SD)	113.84 ± 22.29	116.0 ± 22.15	111.0 ± 22.25	P = 0.0631	100-110

HR, heart rate, CO, cardiac output, SV, stroke volume, SVV, stroke volume variation, SVR, systemic vascular resistance, LVET, left ventricular ejection time, TFC, thoracic fluid content, CFT, corrected flow time, ICON, index of contractility, STR, systolic time ratio, PEP, pre-ejection period.

Reference ranges were based on manufacturer-provided values.

Compared with patients without albuminuria, those with albuminuria had significantly lower SV (52.61 ± 10.91 vs. 55.84 ± 9.80 mL, p=0.0113) and ICON (29.44 ± 9.48 vs. 34.78 ± 10.18, p<0.0001), higher HR, higher SVV, and higher SVR. No significant differences were observed in CO, LVET, TFC, CFT, or STR.

### Multiple linear regression for factors associated with index of contractility

In multiple linear regression analysis adjusting for sex, body mass index, systolic blood pressure, HbA1c, lipid parameters, and estimated glomerular filtration rate (**Table 3**), the presence of albuminuria remained independently associated with lower ICON (p=0.0184). Higher BMI was strongly associated with lower ICON (p<0.0001), while male sex was associated with higher ICON (p=0.0413). Higher HDL-C was also positively associated with ICON (p=0.0206). Triglyceride levels were no longer independently associated with ICON after these adjustments.

**Table 3:** The multiple linear regression analysis of clinical factors associated with index of contractility.

Variable	DF	Parameter Estimate	Standard Error	t Value	Pr > |t|
Intercept	1	58.57673	6.99659	8.37	<.0001
Sex*	1	2.34044	1.14056	2.05	0.0413
BMI	1	-1.02028	0.13600	-7.50	<.0001
Albuminuria	1	-2.67639	1.12776	-2.37	0.0184
LDL-C	1	-0.00743	0.00889	-0.84	0.4036
SBP	1	-0.01500	0.02834	-0.53	0.5970
HDL-C	1	0.11650	0.05000	2.33	0.0206
HbA1c	1	-0.78716	0.47453	-1.66	0.0985
TC	1	0.01581	0.02006	0.79	0.4314
TG	1	-0.00493	0.00424	-1.16	0.2460
eGFR	1	0.04086	0.02232	1.83	0.0684

BMI, body mass index, LDL-C, low density lipoprotein cholesterol, SBP, systolic blood pressure, HDL-C, high density lipoprotein cholesterol, HbA1c, glycated hemoglobin, TC, total cholesterol, TG, triglyceride, eGFR, estimated Glomerular filtration rate.

*Sex was coded as male = 1, female = 0.

### Hospitalization during follow-up

During follow-up, the all-cause medical hospitalization rate was higher in patients with albuminuria than in those without (39.5% vs. 24.5%, p=0.0079). This analysis was exploratory. Cause-specific cardiovascular or heart-failure hospitalization data were not available.

## Discussion

In this prospective cohort of outpatients with T2DM receiving regular clinical care and without clinically recognized cardiovascular disease, albuminuria was independently associated with an altered hemodynamic profile characterized by lower stroke volume and ICON and higher systemic vascular resistance. The association between albuminuria and lower ICON remained significant after multivariable adjustment including sex, body mass index, and major metabolic and renal factors. Higher BMI was also strongly and independently associated with lower ICON, while male sex was associated with higher ICON values.

Albuminuria may reflect systemic vascular abnormalities in addition to renal injury. It has been associated with generalized endothelial and microvascular dysfunction, supporting the concept that urinary albumin leakage may reflect abnormalities in vascular permeability and microvascular integrity beyond the kidney (15). Albuminuria is also independently associated with cardiovascular disease risk, suggesting that it may serve as an indicator of broader vascular injury within the CKM spectrum (16). Recent findings from the TESEO study further demonstrated an independent association between albuminuria and preclinical left ventricular systolic dysfunction assessed by GLS in patients with T2DM (17). Together, these observations provide a biological and clinical context for our findings. Rather than representing an isolated renal abnormality, albuminuria may identify patients with a systemic vascular phenotype in whom early cardiovascular hemodynamic alterations are already present.

The pattern observed in our study, namely reduced SV and ICON accompanied by increased SVR, may reflect alterations in forward flow and cardiac loading conditions. Increased SVR represents greater ventricular afterload, while reduced SV may indicate impaired forward-flow dynamics. The coexistence of these findings in patients without clinically recognized cardiovascular disease suggests that hemodynamic abnormalities may be detectable before the development of symptomatic HF. Because our study was observational, albuminuria may serve as a marker of systemic vascular and metabolic abnormalities that are associated with an altered cardiovascular hemodynamic state.

The physiological basis of electrical cardiometry supports a cautious interpretation of ICON. ICON is derived from the peak amplitude of the rate of change of thoracic impedance (–dZ/dt) relative to baseline impedance, reflecting peak aortic blood acceleration and erythrocyte alignment dynamics during early systole (18, 19). Limited experimental data in animal models have shown a correlation between ICON and end-systolic elastance(a recognized load-independent index of myocardial contractility), particularly under adrenergic stimulation. However, human validation remains limited, and a systematic review and meta-analysis of electrical cardiometry for cardiac output measurement reported acceptable bias but clinically unacceptable mean percentage error (approximately 42–48%), indicating that the technology cannot fully replace thermodilution or echocardiography for absolute values (13). Therefore, reduced ICON in our study should be viewed as a marker of altered hemodynamic phenotype rather than a definitive, load-independent measure of intrinsic myocardial contractility.

The STR, calculated as PEP/LVET via electrical cardiometry, has previously been associated with LVEF; specifically, an STR ≥0.50 was reported to identify LVEF <50% with approximately 90% accuracy in earlier studies (20). In our cohort, the STR values were mostly below 0.50. Nevertheless, the mean ICON was substantially lower than the established reference range. This discrepancy may indicate that conventional systolic indices remain insensitive to early myocardial functional alterations in patients with T2DM. Previous studies have demonstrated that LVEF frequently remains preserved despite the presence of subclinical myocardial dysfunction (21). More sensitive modalities, particularly GLS, have consistently revealed early impairments in myocardial contractile performance before overt reductions in ejection fraction become apparent (8, 22). In this context, the lower ICON observed in our cohort may represent a hemodynamic manifestation of subtle myocardial dysfunction that warrants further investigation with echocardiography and GLS rather than a confirmed diagnosis of subclinical left ventricular dysfunction.

Higher BMI was independently associated with lower ICON values, consistent with previous echocardiographic studies demonstrating that increasing adiposity is associated with subclinical impairment of myocardial systolic function (23, 24). Dyslipidemia and other metabolic disturbances are recognized components of the pathophysiological continuum linking diabetes, vascular dysfunction, and cardiovascular disease (4). The independent associations of albuminuria and BMI with ICON support interpretation of the observed hemodynamic alterations within the broader cardiovascular-kidney-metabolic (CKM) framework.

Our observation of a higher all-cause medical hospitalization rate among patients with albuminuria is consistent with prior evidence. In the Cardiovascular Health Study, albuminuria was associated with increased rates of all-cause and cause-specific hospitalizations in older adults, including circulatory diagnoses (25). The higher rate of all-cause medical hospitalization among patients with albuminuria provides additional clinical context regarding the broader vulnerability of this population. Because cause-specific cardiovascular or heart-failure hospitalization data were unavailable, causal inference remains limited. This hospitalization analysis was exploratory in nature and should not be interpreted as evidence of cardiovascular prognostic significance.

The observed combination of reduced stroke volume and increased systemic vascular resistance may reflect altered cardiac loading and forward-flow dynamics. Impedance cardiography studies have shown that SGLT2 inhibitors can increase stroke volume and ICON while reducing systemic vascular resistance in patients with T2DM (26). Although therapies with established cardiorenal benefits may be clinically relevant in patients with elevated CKM risk, treatment effects were not evaluated in the present study.

Several limitations should be acknowledged. First, this study was conducted in a single center with a relatively modest sample size which may affect the statistical power and precision of the results. Second, the study lacked concurrent echocardiographic or GLS comparisons; although the STR remained within normal limits, this provides only indirect evidence of preserved LVEF and cannot exclude early longitudinal strain impairment. Third, we did not have complete data on baseline use of SGLT2 inhibitors, ACE inhibitors/ARBs, or beta-blockers, which may influence hemodynamic parameters. Fourth, the absolute accuracy and precision of electrical cardiometry for contractility indices remain limited according to available meta-analyses. Finally, hospitalization data was exploratory and based on medical (non-surgical, non-trauma) admissions without detailed cause-specific or time-to-event analysis.

## Conclusions

Our study demonstrates an association between albuminuria and an altered hemodynamic phenotype (reduced stroke volume and ICON, elevated systemic vascular resistance) in outpatients with T2DM receiving regular clinical care, as assessed by portable noninvasive cardiometry. These findings support further investigation of point-of-care hemodynamic assessment for identifying high-risk CKM phenotypes. Prospective studies incorporating established cardiac imaging and cardiovascular outcomes are needed to validate these observations.

## Data Availability

The raw data supporting the conclusions of this article will be made available by the authors, without undue reservation.
